# Aerobic exercise improves endothelial function and serum adropin levels in obese adolescents independent of body weight loss

**DOI:** 10.1038/s41598-017-18086-3

**Published:** 2017-12-18

**Authors:** Hao Zhang, Long Jiang, Yu-Jing Yang, Ren-Kai Ge, Ming Zhou, Huan Hu, Hui Liu, Jie Cui, Le-Liang Li, Yi-Fei Dong, Xiao-Shu Cheng, Rong Chen, Ping Li

**Affiliations:** 1 Department of Cardiovascular Medicine, the Second Affiliated Hospital of Nanchang University, Nanchang, 330006 Jiangxi Province China; 2 School of Physical Education, East China Jiaotong University, Sports Fitness Research Center, Nanchang, 330013 Jiangxi Province China; 3 Department of Cardiovascular Medicine, Xiangdong Hospital Affiliated to Hunan Normal University, Liling, 412200 Hunan Province China

## Abstract

Adropin is a secreted protein that regulates endothelial function. However, adropin levels in obese adolescent patients are currently uncertain. Therefore, we evaluated the association between plasma adropin levels and vascular endothelial function and investigated the effect of aerobic exercise in obese adolescents. A total of 45 obese adolescents and 20 controls (age 16–19 years) were included in our study. The obese adolescents received 12 weeks of aerobic exercise training. Serum adropin was detected using enzyme-linked immunosorbent assay. Vascular reactive hyperemia indexes (RHIs) were obtained using Endo-PAT2000. Adropin levels and RHI were significantly lower in obese adolescents than in normal-weight adolescents. Adropin levels and RHI increased significantly independently of changes in body weight after an exercise intervention (P < 0.01). Pearson correlation analysis revealed that adropin levels positively correlated with HDL-C levels (r = 0.389, P < 0.01) and RHI (r = 0.32, P < 0.01). Multiple linear stepwise regression analysis showed that the insulin resistance index (t = −3.301, P < 0.01) and HDL-C level (t = 2.620, P = 0.011) were independent risk factors of adropin levels. In addition, Δadropin (t = 3.261, P < 0.01) was an independent influencing factor of ΔRHI. Our findings suggest that adropin plays an important role in vascular endothelial function in obese adolescents.

## Introduction

Obesity is a mild systemic inflammatory disease that is a major risk factor for hypertension, diabetes, coronary heart disease and other chronic diseases^1^. Adolescent obesity has attracted attention worldwide in recent years because of rapid population growth^2^. Obesity causes chronic injury to the cardiovascular, respiratory and endocrine systems of adolescents and lasting harm into adulthood, such as the development of hypertension, diabetes, coronary heart disease and other diseases^1^. Adult cardiovascular disease risk of death is also significantly higher in obese groups^3^. The main treatment for obese adolescents is lifestyle intervention, and aerobic exercise intervention is also important. There are significant differences in blood lipids, insulin resistance and inflammatory factors between obese adolescents and normal-weight adolescents^4^. Aerobic exercise intervention significantly reduces blood lipids, insulin resistance and inflammatory factors in obese adolescents^5^.

Adropin is a novel secreted protein that was found in 2008 by Kumar in the study of energy metabolism in mice, and it regulates insulin sensitivity and the balance of energy metabolism^6^. Some studies reported that low adropin levels are a risk factor of coronary heart disease and in-stent restenosis, and the adropin concentration gradually decreases with increasing severity and lesions of coronary artery disease^7^. One study suggested adropin as a new potential biomarker of acute myocardial infarction^8^. In addition, a study using mouse endothelial cells found that adropin activated the transduction pathway of endothelial nitric oxide synthase (eNOS), which regulates vascular endothelial function and plays an important role in vascular protection^9^. Japanese scholars reported that exercise intervention significantly elevated serum adropin levels in elderly subjects and suggested changes in serum adropin concentration as one possible mechanism of exercise intervention to improve arterial stiffness^10^. However, whether exercise intervention affects serum adropin levels in obese adolescents is not clear.

The present study included 45 obese adolescents and 20 adolescents with normal weight and observed the differences in clinical indices between obese and normal-weight adolescents. The obese adolescent group performed 12 weeks of aerobic exercise, and changes in body composition, lipid metabolism, vascular endothelial function and the serum adropin concentration from before to after the exercise intervention were examined. The present study investigated the effect of an exercise intervention on lipid metabolism, vascular endothelial function and serum adropin levels in obese adolescents to provide a novel way of thinking in the prevention of long-term risk for cardiovascular disease in obese adolescents.

## Methods

### Candidates

This study recruited 50 young obese adolescents (aged between 16–19 years old) in East China Jiaotong University and 30 normal-weight adolescents as a control group. All participants signed informed consent forms and were supported by their legal guardians before study inclusion. A total of 45 obese and 20 normal-weight adolescents were included. The research ethics committees of the Second Affiliated Hospital of Nanchang University and East China Jiaotong University approved this research. We confirm that all methods were performed in accordance with the relevant guidelines and regulations.

### Inclusion Criteria

All participants were freshmen from East China Jiaotong University. The inclusion criteria referred to the reference norm of body mass index (BMI) for the screening of overweight and obesity in Chinese children and adolescents from the China Obesity Task Force in 2004^11^. The following inclusion criteria were used. (1) Among 16–17-year-olds, males with BMI < 23.5 kg/m^2^ were classified as normal weight, and males with BMI ≥ 27.4 kg/m^2^ were classified as obese; Females with BMI < 23.7 kg/m^2^ were classified as normal weight, and females with BMI ≥ 27.4 kg/m^2^ were classified as obese. (2) Among 17–18-year-olds, males with BMI < 23.8 kg/m^2^ were classified as normal weight, and males with BMI ≥ 27.8 kg/m^2^ were classified as obese; females with BMI < 23.8 kg/m^2^ were classified as normal weight, and obese female BMI ≥ 27.7 kg/m^2^ were classified as obese. (3) Among subjects over 18 years of age, males and females with BMI < 24.0 kg/m^2^ were classified as normal weight, and males and females with ≥ 28 kg/m^2^ were classified as obese.

### Exclusion criteria

Subjects with secondary obesity, a medical history of cardiovascular disease, respiratory system disease, kidney disease, a history of smoking, a history of use of any hormone or weight-reducing drugs, or a history of habitual physical activity were excluded from the study.

### Exercise program

We organized health promotion classes with professional sports teachers based on the platform of the College of Physical Education of East China Jiaotong University. Obese adolescents in the health promotion class received 12 weeks of aerobic exercise. The project gave priority to jogging and assisted in other sports, including badminton, table tennis, aerobics and cycling^12,13^. Heart rate was used as the monitoring indicator of exercise intensity. Exercise intensity was controlled at 60–80% of the maximum heart rate using a Type 750 telemetering heart rate monitoring instrument (POLAR, Finland)^14^. Peak VO2 was defined as the highest VO2 that could be sustained for at least 30 s during the last stage of cycling exercise. Peak oxygen uptake = Peak VO2. Peak VO2 was measured during breath-by-breath assessment using an incremental cycle exercise test on a cycle ergometer (MINATO, AE-310SRD, Osaka, Japan)^14^. All candidates trained 3–5 times per week (mean and standard deviation: 3.67 ± 0.78 sessions) for 90 min per session under the guidance of professional sports coaches. The obese group completed 90% of the program during the course of 12 weeks of exercise. The details of the exercise program are described in previous studies^12,13^.

### Measurement of body morphology index

Height, weight, waist circumference, hip circumference and blood biochemical tests were measured in all subjects one day before initiation of the aerobic exercise training program. Trained and qualified personnel performed all data measurements, and two people supervised each other during the measurements and recordings to ensure data accuracy. The TBF-41B body fat analyzer (Bailida Health Equipment Co., Ltd., China) was used to measure the body composition of all research subjects. The above physical fitness indices were measured in obese adolescents after the 12 weeks of training. The measuring instruments, methods and personnel were consistent with the first measurement. Waist-to-hip ratio (WHR) = waist / hip circumference, and BMI = weight (kg)/height^2^(m^2^).

### Blood biochemical tests

Blood samples were collected from all subjects one day prior to the aerobic intervention. None of the female subjects were in their menstrual period at the time of blood sample collection. Blood samples were collected between 7 and 9 a.m. after 12 hours of fasting and sent immediately to the laboratory of the Second Affiliated Hospital of Nanchang University. The following parameters were tested: liver and kidney function as well as the levels of triglyceride (TG), total cholesterol (TC), high-density lipoprotein cholesterol (HDL-C), low-density lipoprotein cholesterol (LDL-C), fasting glucose (Glu), and fasting insulin (FINS). The insulin resistance index (HOMA-IR) was calculated as follows: HOMA-IR = FBG*FINS /22.5. A second series of blood samples were collected from the obese group two days after the 12-week aerobic exercise program. The requirements of blood samples, testing indicators, testing instruments and testing units were identical to those of the first collection.

### Plasma adropin measurements

Adropin concentrations were measured using an adropin (ENHO) ELISA reagent kit (Cusabio Biotech Co., Ltd., Wuhan, China). The detection limits ranged from 1.56 to 100 pg/ml, and the intra-assay coefficient of variation was <8%.

### Detection of blood pressure and vascular endothelial function

Blood pressure and vascular endothelial function tests were completed within one week before and after 12 weeks exercise training. Blood pressure measurements were obtained using an OMRON electronic blood pressure monitor (HEM-7112, Japan). The process of measuring blood pressure was performed according to the 2010 “Chinese Blood Pressure Measurement Guidelines”^15^. Measurement of vascular endothelial function: Fasting endothelial function was measured using Endo-PAT 2000 (Itamar Medical Ltd., Caesarea, Israel) every morning. Subjects were asked to avoid strenuous exercise, alcohol, tobacco, tea and coffee, which may influence the measurement of blood pressure, 12 hours before measurement. Subjects remained quiet and relaxed and sat in a comfortable position during the strictly performed testing protocol. First, subjects placed their index finger in the Endo-PAT biosensor probe to detect vascular endothelial function on one side. The other side was the control that responded to dynamic changes in whole blood vessels. A standard cuff (Hokanson AG101, D. E. Hokanson Inc., Bellevue, Was) was placed on the upper 2 cm of the brachial artery without pneumatic compression. Second, a 1-min signal and stability test was performed, and baseline tension data were then collected for 5 min using a fast-filling gas at the end of the cuff (general pressure to 200 mmHg or higher shrinkage pressure of 60 mmHg). The cuff was quickly deflated, and blood flow signal acquisition was processed for 5 minutes. The total measurement process lasted 16 minutes. Finally, Endo-PAT 2000 computer software (Medical Itamar) automatically collected signal analyses to obtain the reaction of vascular endothelial function of the vascular reactive hyperemia index (RHI)^16^.

### Statistical analysis

Data were statistically analyzed using SPSS 19.0 software (SPSS Inc., USA). Data are reported as the means ± standard deviation for quantitative variables and percentages for qualitative variables. Between-group (obese and normal-weight groups) comparisons were performed using independent-sample t-tests (2-tailed) and chi-square tests. The comparison of data between before and after the exercise intervention in the obese group was conducted using a paired-sample t-test. Correlation analysis was performed using Pearson correlation and multiple linear stepwise regression analysis. P < 0.05 indicated that the difference was statistically significant.

## Results

### The clinical baseline

There were no differences in age, sex, or height between the obese and control groups (Table 1). The obese group exhibited higher BMI, WHR, fat mass, aspartate aminotransferase (AST) levels, uric acid levels, systolic blood pressure (SBP), and diastolic blood pressure (DBP) than the control group [SBP: 128.7 ± 13.6 mmHg vs. 113.7 ± 10.7 mmHg, P < 0.001; DBP: 69.6 ± 7.9 mmHg vs. 61.3 ± 6.5 mmHg, P < 0.001]. In addition, 15.6% of the obese adolescents were diagnosed as hypertensive. The obese group exhibited higher TG, fasting blood glucose and fasting insulin levels and HOMA-IR than the control group (Table 1). A total of 28.9% of the obese group exhibited higher lipid levels and 66.7% had higher HOMA-IR based on the normal reference value^17,18^. However, the HDL-C level was significantly lower in the obese group. The obese group exhibited significantly lower serum adropin levels [(2.64 ± 0.93) ng/ml vs. (3.23 ± 0.87) ng/ml, P = 0.018] and RHI [(1.70 ± 0.37) vs. (2.03 ± 0.43), P = 0.002] than the control group (Figure S1).

**Table 1:** Baseline characteristics of the obese and control groups.

	Obese Group (n = 45)	Control Group (n = 20)	P value
Age (years)	17.9 ± 0.8	18.0 ± 0.7	0.521
Male (n, %)	36 (80.0%)	13 (65.0%)	0.325
SBP (mmHg)	128.7 ± 13.6	113.7 ± 10.7	<0.001**
DBP (mmHg)	69.5 ± 7.9	61.3 ± 6.5	<0.001**
Height (cm)	168.3 ± 6.7	165.2 ± 9.2	0.184
Weight (kg)	88.9 ± 8.7	63.2 ± 11.2	<0.001**
WHR	0.91 ± 0.04	0.84 ± 0.05	<0.001**
BMI (kg/m^2^)	31.4 ± 2.7	20.4 ± 2.6	<0.001**
Fat mass (kg)	28.1 ± 6.1	13.9 ± 4.5	<0.001**
Total bilirubin (μmol/l)	12.1 ± 4.6	11.4 ± 3.3	0.526
AST (μmol/l /l)	32.4 ± 20.2	20.2 ± 7.4	0.001**
ALT (μmol/l /l)	22.6 ± 7.6	20.5 ± 5.4	0.278
BUN (mmol/l)	4.2 ± 1.0	4.3 ± 1.3	0.543
Creatinine (μmol/l)	74.4 ± 11.0	71.4 ± 11.0	0.299
Uric acid (μmol/l /l)	438.0 ± 89.7	363.6 ± 70.7	0.002**
TC (mmol/l)	4.14 ± 0.65	4.03 ± 0.69	0.562
TG (mmol/l)	1.33 ± 0.66	0.94 ± 0.34	0.003**
HDL-C (mmol/l)	1.09 ± 0.18	1.28 ± 0.33	0.005**
LDL-C (mmol/l)	2.49 ± 0.59	2.29 ± 0.58	0.198
Glu (mmol/l)	4.94 ± 0.49	4.67 ± 0.47	0.042*
Fasting insulin (μUI/ml)	22.0 ± 8.3	12.4 ± 6.2	<0.001**
HOMA-IR	4.8 ± 1.9	2.6 ± 1.5	<0.001**
Adropin (ng/ml)	2.64 ± 0.93	3.23 ± 0.87	0.018*
RHI	1.70 ± 0.37	2.03 ± 0.43	0.002**
Peak VO2, ml/kg/min	26.7 ± 1.8	32.8 ± 1.4	< 0.001**

Note: AST: aspartate aminotransferase; ALT: alanine aminotransferase; BMI: body mass index; BUN: blood urea nitrogen; DBP: diastolic blood pressure; Glu: fasting glucose; HDL-C: high-density lipoprotein cholesterol; HOMA-IR: insulin resistance index; LDL-C: low-density lipoprotein cholesterol; RHI: reactive hyperemia index; SBP: systolic blood pressure; TC: total cholesterol; TG: triglyceride; WHR**:** waist-to-hip ratio. Data are presented as the means ± standard deviation, *P < 0.05, **P < 0.01.

### Effect of aerobic exercise in the obese group

**Figure 1:**
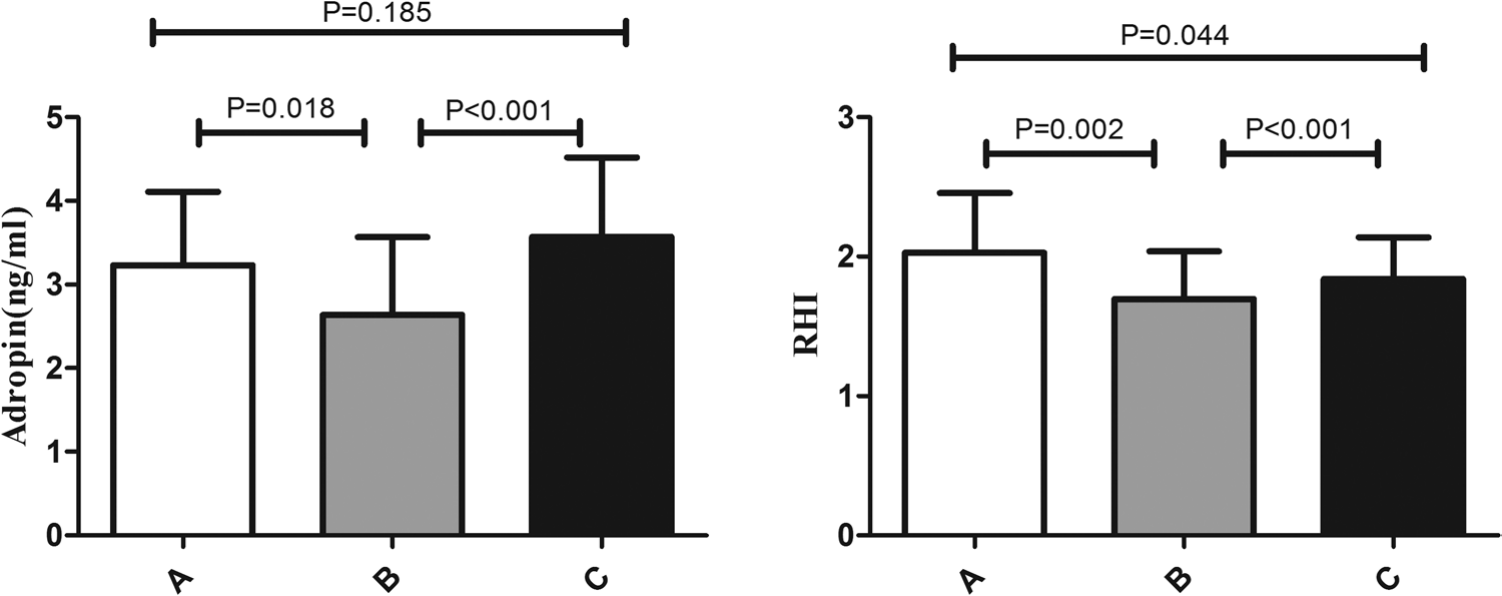
Comparison of the serum adropin concentration and vascular reactive hyperemia index (RHI) between before and after intervention in all subjects. (**A**) Control group. (**B**) Obese group before the intervention. (**C**) Obese group after the intervention. Before the intervention, adropin levels and RHI were lower in the obese group than in the control group. After the intervention, adropin levels and RHI were higher than before the intervention in the obese group.

Twelve weeks of aerobic exercise training significantly decreased body morphology indices, like weight, BMI, waist circumference, WHR, and fat mass, in the obese group (Table S1). SBP also decreased [(128.7 ± 13.6) mmHg vs. (123.4 ± 12.3) mmHg, P < 0.01],but DBP was not different after the exercise intervention [(69.8 ± 7.9) mmHg vs. (70.3 ± 8.7) mmHg, P = 0.601]. Blood pressure was restored to normal levels in nearly half of the obese adolescents who exhibited abnormal blood pressure prior to exercise. ALT, blood urea nitrogen (BUN), and creatinine levels were significantly reduced after exercise intervention. TC and LDL-C levels were significantly reduced, and HDL-C was increased compared to baseline levels. The obese group exhibited significantly deceased Glu, fasting insulin, and HOMA-IR after 12 weeks of aerobic exercise (Table S1). Notably, serum adropin levels [(2.64 ± 0.93) ng vs. (3.57 ± 0.95) ng, P < 0.01] and RHI [(1.70 ± 0.37) vs. (1.84 ± 0.29), P < 0.01] were significantly increased after exercise compared to before training in obese subjects (Fig. 1).

### Subgroup analysis of the obese group based on weight changes after exercise intervention

The obese group was divided into a weight loss group (n = 37) and a weight gain group (n = 8) based on the changes in weight. The clinical parameters of before and after aerobic exercise were compared in either weight loss group or weight gain group. In weight loss group, there is a significant decrease in AST (P < 0.001), LDL-C (P < 0.001) and Glu levels (P < 0.001) after 12 weeks of training compared before intervention (Table 2). Notably, the levels of HDL-C (P < 0.001), serum adropin (P < 0.001), and RHI (P < 0.001) were also significantly increased after the intervention (Table 2). In weight gain group, the results were the same as the weight loss group. There is a significant decrease in AST (P = 0.042), LDL-C (P < 0.001) and Glu levels (P < 0.001) after 12 weeks of training and the levels of HDL-C (P < 0.001), serum adropin (P = 0.004), and RHI (P = 0.02) were significantly increased after the intervention (Table S2).

**Table 2:** Comparison of parameters between before and after exercise intervention in the weight loss group.

	Before intervention (n = 37)	After intervention (n = 37)	P value
Male (n, %)	33(89.2%)	33(89.2%)	—
Height (cm)	168.9 ± 6.6	169.2 ± 6.7	0.024
Weight (kg)	88.9 ± 9.2	83.1 ± 10.4	<0.001**
BMI (kg/m2)	31.2 ± 2.6	29.0 ± 2.9	<0.001**
SBP (mmHg)	129.1 ± 14.2	123.8 ± 12.9	0.003**
DBP (mmHg)	69.4 ± 8.0	69.9 ± 9.1	0.727
Fat mass (kg)	27.2 ± 5.1	19.8 ± 5.0	<0.001**
Total bilirubin (μmol/l)	12.5 ± 4.8	13.6 ± 6.0	0.141
AST (μmol/l /l)	33.9 ± 21.2	23.9 ± 22.7	<0.001**
ALT (μmol/l /l)	22.9 ± 7.8	21.4 ± 10.8	0.299
BUN (mmol/l)	4.1 ± 1.0	3.7 ± 0.9	0.002**
Creatinine (μmol/l)	75.2 ± 11.0	72.0 ± 11.6	0.001**
Uric acid (μmol/l /l)	440.8 ± 94.2	432.0 ± 102.2	0.395
TC (mmol/l)	4.23 ± 0.64	3.93 ± 0.58	<0.001**
TG (mmol/l)	1.30 ± 0.62	1.28 ± 0.52	0.782
HDL-C (mmol/l)	1.08 ± 0.18	1.26 ± 0.23	<0.001**
LDL-C (mmol/l)	2.61 ± 0.57	2.19 ± 0.44	<0.001**
Glu (mmol/l)	4.95 ± 0.51	4.15 ± 0.48	<0.001**
Fasting insulin (μUI/ml)	21.6 ± 8.1	15.9 ± 7.0	<0.001**
HOMA-IR	4.8 ± 1.9	3.0 ± 1.5	<0.001**
Adropin (ng/ml)	2.62 ± 0.87	3.45 ± 0.65	<0.001**
RHI	1.71 ± 0.40	1.86 ± 0.31	<0.001**
VO2peak,ml/kg/min	25.8 ± 1.6	29.2 ± 1.4	<0.05*

Note: AST: aspartate aminotransferase; ALT: alanine aminotransferase; BUN: blood urea nitrogen; DBP: diastolic blood pressure; Glu: fasting glucose; HDL-C: high-density lipoprotein cholesterol; HOMA-IR: insulin resistance index; LDL-C: low-density lipoprotein cholesterol; RHI: reactive hyperemia index; SBP: systolic blood pressure; TC: total cholesterol; TG: triglyceride; data are presented as the means ± standard deviation, *P < 0.05, **P < 0.01.

### Correlation analysis of serum adropin level with clinical parameters

**Figure 2:**
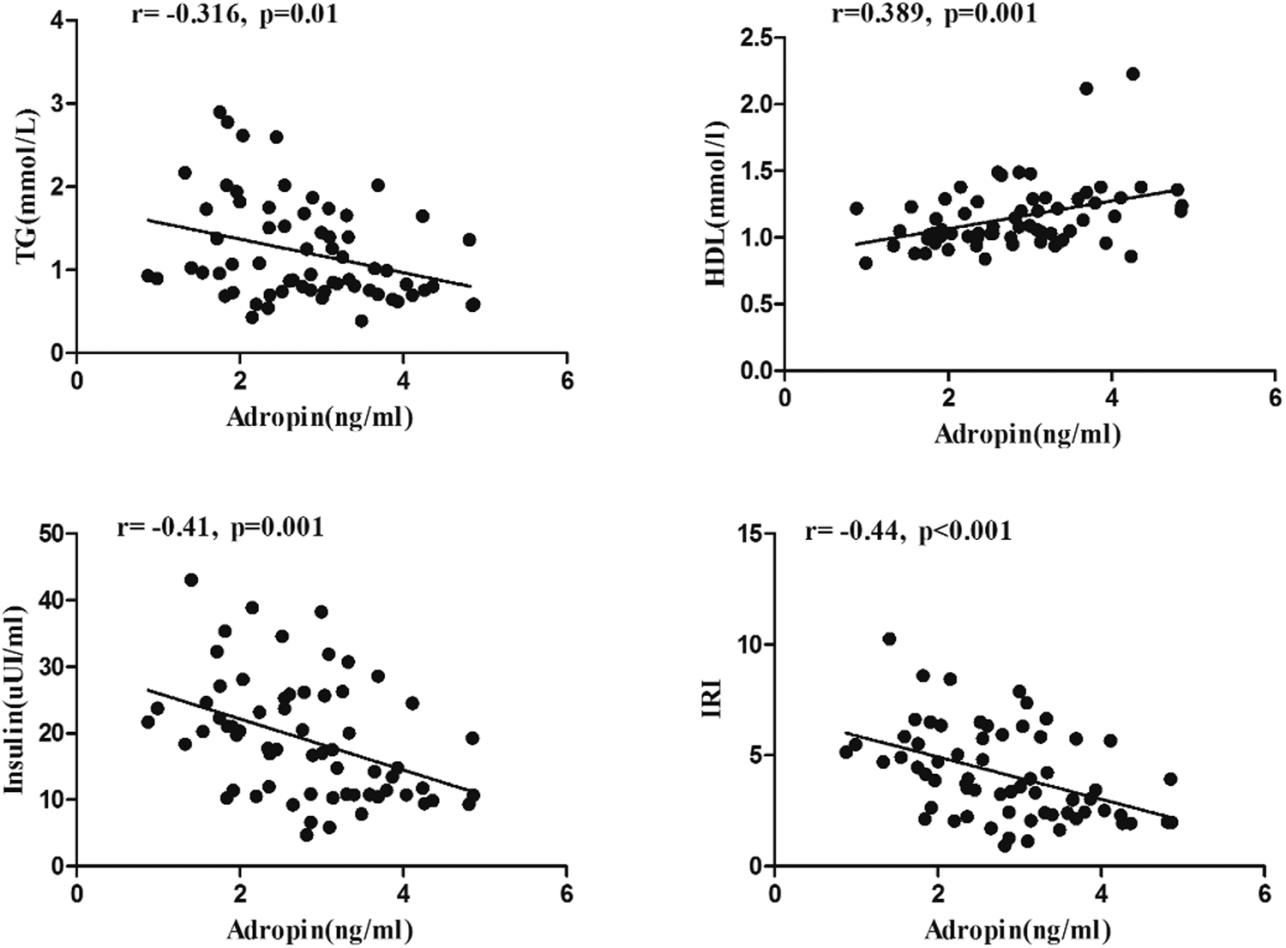
The Pearson correlation analyses of the serum adropin level with TG, HDL-C and insulin levels as well as RHI in all subjects. There was a negative relationship between adropin levels and TG levels, insulin levels, and RHI and a positive relationship between adropin levels and HDL-C levels.

Pearson correlation analysis in all subjects demonstrated that the serum adropin level positively correlated with the HDL-C level (r = 0.389, P < 0.01) and RHI (r = 0.32, P < 0.01) (Table S3). In contrast, serum adropin levels negatively correlated with DBP (r = −0.272, P < 0.05), weight (r = −0.35, P < 0.01), BMI (r = −0.248, P < 0.05), WHR (r = −0.335, P < 0.01), AST levels (r = −0.313, P < 0.05), uric acid levels (r = −0.424, P < 0.01), TG levels (r = −0.316, P = 0.01), Glu levels (r = −0.266, P < 0.05), fasting insulin levels (r = −0.41, P < 0.01), and HOMA-IR (r = −0.44, P < 0.01) (Fig. 2). Pearson analysis of each group revealed that the relationships were primarily from the obese group (Figs S2 and S3).

Multiple linear regression analysis demonstrated that HOMA-IR (t = −3.301, P < 0.01) and HDL-C (t = 2.620, P = 0.011) were the independent influencing factors of the serum adropin concentration (Table S4).

### Correlation analysis of ΔRHI with quantitative changes in other clinical parameters in the obese group

**Figure 3:**
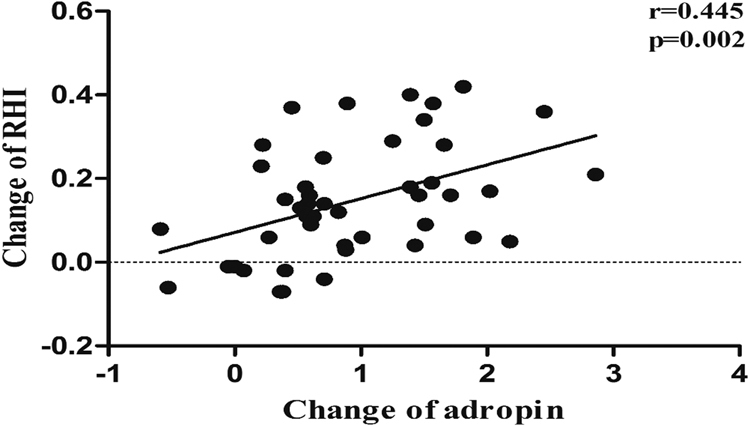
The correlation analysis of the change in RHI with the change in serum adropin levels from before to after the exercise intervention in obese adolescents. The results demonstrated that ΔRHI positively correlated with Δadropin.

For all parameters, Δ was defined as the amount of change from before to after exercise. Pearson correlation analysis revealed that ΔRHI positively correlated with ΔSBP (r = 0.317, P < 0.05), Δcreatinine (r = 0.322, P < 0.05), and Δadropin (r = 0.445, P < 0.01) (Fig. 3 and Supplement Table S5) in the obese group. Multivariate linear regression analysis revealed that only Δadropin (t = 3.261, P < 0.01) was an independent influencing factor of ΔRHI.

## Discussion

Lancet published a research report on overweight or obese in 188 countries in June 2014^19^. This study demonstrated that the total number of overweight and obese people worldwide increased from 857 million in 1980 to 2.1 billion in 2013. The China Disease Prevention Center reported that 12% of Chinese children and adolescents were overweight (approximately 120 million), which suggests that obesity was a serious public health problem in China. Tirosh *et al*. published a large prospective study of 37,647 adolescents with an average of 17.4 years of follow-up. They found that adolescent obesity was an independent risk of coronary heart disease and diabetes^20^. Obese adolescents also exhibited early endothelial dysfunction, and there was a significant relationship between vascular endothelial dysfunction and obesity^21,22^.

The primary treatment for obese adolescents is lifestyle intervention, and aerobic exercise training is an important component. Our results demonstrated that body weight, BMI, waist circumference, WHR, body fat mass and body fat percentage decreased significantly after 12 weeks of aerobic exercise. These results demonstrated that aerobic exercise intervention significantly improved obesity status in obese adolescents. In the obese group, 15.6% of the subjects were diagnosed with high blood pressure, and 12 weeks of exercise significantly reduced their SBP. In addition, 28.9% of obese adolescents exhibited abnormal blood lipid levels. After the 12-week aerobic exercise intervention, 80% of the obese adolescents with abnormal blood lipid levels exhibited a return to normal lipid levels. Our data suggest that 66.7% of obese adolescents exhibited an elevated insulin resistance index at study inclusion before exercise. The insulin resistance index was significantly reduced in obese adolescents after 12 weeks of aerobic exercise intervention. Aerobic exercise improves human body fat metabolic enzyme activity, accelerates fat decomposition and utilization, and effectively inhibits fat synthesis^23^. Therefore, a suitable aerobic exercise intervention gradually eliminates fat accumulation in the body and improves glucolipid metabolism and insulin resistance^24^. Insulin resistance exhibits a significant positive correlation with long-term cardiovascular events^25^. Improvement of insulin resistance status using an early exercise intervention may be of great significance for reducing future cardiovascular events in obese adolescents.

Adropin is a newly identified stable secretory protein that contains 76 amino acids, and it is encoded by a protein energy homeostasis-related gene (Enho)^6^. Adropin is expressed in liver, brain, umbilical vein and coronary artery endothelial cells^6^. Serum adropin levels are significantly lower in obese children^26^, and a low adropin level is a risk factor for coronary heart disease, hypertension, and metabolic syndrome^27–29^. Our study found that serum adropin levels were significantly lower in obese adolescents than in normal-weight adolescents, and Pearson correlation analysis revealed that the serum level of adropin negatively correlated with BMI (r = −0.248, P < 0.05). Twelve weeks of aerobic exercise intervention significantly increased the serum level of adropin in obese adolescents, regardless of weight gain or weight loss in this group. This result indicated that the increase in serum adropin concentration was a direct benefit of 12 weeks of aerobic exercise itself and occurred independent of changes in body weight. However, the mechanism of the exercise-induced increase in serum adropin level is not clear. Further studies are needed to confirm the role of adropin in the regulation of glucolipid metabolism.

Previous research has shown that adropin plays a role in glucose and lipid metabolic disturbances^6^. Our study confirmed that serum adropin levels negatively correlated with the levels of TG, fasting glucose, and insulin as well as insulin resistance and positively correlated with HDL-C levels. Previous studies reported that adropin in muscle cells inhibited silent information regulator 1 (SIRT1)^30^, which regulates the activity of peroxisome proliferator-activated receptor gamma-activating factor 1 alpha (PGC-1a)^31^. PGC-1a affects the activity of carnitine palmitoyltransterase-1 (Cpt1) and pyruvate dehydrogenase kinase (Pdk4), which play key roles in the oxidation of fatty acids and glucose oxidation via PDK pathway^32,33^. Butler *et al*. included 130 volunteers and found significant negative associations between plasma adropin levels and TG, ApoB, LDL-C, glucose, and blood pressure and positive associations between plasma adropin and HDL-C^34^. These results suggest a close relationship between adropin and lipid and glucose metabolism. Adropin may be an effective intervention target for improving dyslipidemia and insulin resistance in patients with diabetes mellitus.

Vascular endothelial dysfunction is an early manifestation of cardiovascular disease, but it is a reversible process^21^. Therefore, preventing the occurrence of cardiovascular events following the early detection of vascular endothelial dysfunction and effective intervention is very important. Several studies confirmed that aerobic exercise intervention was an effective strategy to improve vascular endothelial dysfunction in obese adolescents^22^. Endo-PAT2000 detects fingertip vascular volume pressure caused by changes in the arterial pulse and transforms these changes into peripheral arterial tension to calculate the RHI response to vascular endothelial function using specialized computer software. However, whether the exercise intervention influenced the RHI of obese children was not known. Our research demonstrated a significant increase in RHI in obese adolescents (1.84 + 0.29 vs 1.70 + 0.37, P < 0.01) after a 12-week exercise intervention in the weight gain and weight loss subgroups, which suggests that exercise training significantly and directly improved vascular endothelial function in obese adolescents. The change in RHI also occurred independent of changes in body weight. Our study demonstrated that 12 weeks of aerobic exercise partially reversed vascular endothelial dysfunction related to obesity. The following possible mechanisms may underlie these observations: (1) exercise enhances endothelial nitric oxide synthase function and promotes the vasodilator release of cytokines; (2) exercise improves insulin sensitivity, reduces inflammation and improves lipoprotein dysfunction^22^. Further research is needed to demonstrate the effect and pathophysiological mechanisms of exercise intervention on the RHI in obese adolescents.

Adropin may be a novel and effective serum marker for the evaluation of endothelial function^35^. Our study also found a positive correlation between the change in RHI and the changes in systolic blood pressure, serum creatinine levels, and the serum concentration of adropin. Multiple linear regression analysis demonstrated that only the change in the serum adropin concentration change was an independent factor influencing the change in RHI. This result suggests that the change in the serum adropin concentration may be one mechanism by which the exercise intervention improved vascular endothelial function in obese adolescents. Previous studies also demonstrated that a lower serum adropin level was associated with endothelial dysfunction using flow-mediated dilatation in patients with type-2 diabetes mellitus and metabolic syndrome^36,37^. Aerobic exercise training increased the serum adropin level and improved arterial stiffness and adiposity in obese adults^38^. The following mechanisms of the beneficial effects of adropin on endothelial function are proposed. (1) Serum adropin promotes the production of nitric oxide and increases nitric oxide bioavailability, which improves arterial stiffness^10^. (2) Adropin activates signal transduction in endothelial cells, potentially through two pathways: the vascular endothelial growth factor 2 (VEGFR2)-PI3-Akt pathway and the VEGFR2-extracellular signal-regulated kinase 1/2 (ERK1/2) pathway; such signal transduction increases eNOS expression, which increases NO synthesis^9^. Our results suggest serum adropin as a possible mechanism underlying the aerobic exercise-induced improvement of endothelial function.

### Limitations

There are some limitations of our study. First, the sample size of this study was small, and the most of the subjects were male. Gender differences may impact the research data. Second, we did not include an obese control group during exercise intervention because of ethical factors, which is also a deficiency of this study. Third, the time span of our study was 3 months, from Spring to Summer, and seasonal changes may influence blood pressure, blood vessel endothelial function and other indicators. Finally, our research was a clinical study, and the related mechanisms of our results should be further elucidated.

## Conclusion

Our study demonstrated that 12 weeks of exercise intervention increased the serum level of adropin and improved endothelial function. This result suggests an important role for adropin in vascular endothelial function in obese adolescents.

## Electronic supplementary material

Supplementary Information
