# Supplementary material for: Aerobic exercise improves endothelial function and serum adropin levels in obese adolescents independent of body weight loss
Source: Sci Rep. 2017 Dec 18;7:17717. doi: 10.1038/s41598-017-18086-3 (PMC5735148; doi:10.1038/s41598-017-18086-3)
Supplement: Supplementary file 1 — Supplementary Information [file 41598_2017_18086_MOESM1_ESM.doc]

**Aerobic exercise improves endothelial function and serum adropin level in obese adolescents independent of body weight loss**

Hao Zhang#^1^, Long Jiang#^1^, Yu-Jing Yang^1^, Ren-Kai Ge^2^, Ming Zhou^2^, Huan Hu^1^, Hui Liu^1^, Jie Cui^1^, Le-Liang Li^1^, Yi-Fei Dong^1^, Xiao-Shu Cheng^1^, Rong Chen*^2^, Ping Li*^1^

**Supplemental**

**Table S1 Comparison of various indexes of obesity group before and after exercise intervention**

**Table S2 Comparison of parameters between before and after exercise intervention in the weight gain group**

**Table S3. The Pearson correlation analysis of serum adropin level and the clinical data**

**Table S4.** **Results of multiple linear regression analysis of serum adropin**

**Table S5 Pearson related analysis results of ΔRHI and clinical data in obese adolescents before and after exercise intervention**

**Figure S1 Comparison of serum adropin concentration and vascular reactive congestion index (RHI) in normal weight group and obesity group**

**Figure S2 The pearson correlation analysis of serum adropin level and TG, HDL-C, insulin, and RHI in control group**

**Figure S3 The pearson correlation analysis of serum adropin level and TG, HDL-C, insulin, and RHI in obesity group**

Table S1 Comparison of various indexes of obesity group before and after exercise intervention

|  | Before intervention  (n=45) | After intervention  (n=45) | P value |
| --- | --- | --- | --- |
| SBP (mmHg) | 128.7 ± 13.6 | 123.4 ± 12.3 | 0.001** |
| DBP (mmHg) | 69.8 ± 7.9 | 70.3 ± 8.7 | 0.601 |
| Height (cm) | 168.3 ± 6.7 | 168.7 ± 6.8 | 0.005** |
| Weight (kg) | 88.9 ± 8.7 | 84.3 ± 10.2 | <0.001** |
| WHR | 0.91 ± 0.04 | 0.89 ± 0.05 | <0.001** |
| BMI (kg/m^2^) | 31.4 ± 2.7 | 29.6 ± 3.2 | <0.001** |
| Fat mass (kg) | 28.1 ± 6.1 | 21.6 ± 7.5 | <0.001** |
| Total bilirubin (μmol/l) | 12.1 ± 4.6 | 12.9 ± 5.9 | 0.248 |
| AST (μmol/l /l) | 32.4 ± 20.2 | 23.2 ± 21.1 | <0.001** |
| ALT (μmol/l /l) | 22.6 ± 7.6 | 20.9 ± 10.0 | 0.180 |
| BUN (mmol/l) | 4.2 ± 1.0 | 3.8 ± 1.0 | 0.001** |
| Creatinine (μmol/l ) | 74.4 ± 11.0 | 71.5 ± 12.1 | 0.002** |
| Uric acid (μmol/l /l) | 438.0 ± 89.7 | 431.8 ± 99.5 | 0.503 |
| TC (mmol/l) | 4.14 ± 0.65 | 3.88 ± 0.58 | <0.001 |
| TG (mmol/l) | 1.33 ± 0.66 | 1.33 ± 0.69 | 0.936 |
| HDL-C (mmol/l) | 1.09 ± 0.18 | 1.30 ± 0.24 | <0.001** |
| LDL-C (mmol/l) | 2.49 ± 0.59 | 2.11 ± 0.44 | <0.001** |
| Glu (mmol/l) | 4.94 ± 0.49 | 4.15 ± 0.46 | <0.001** |
| Fasting insulin (μUI/ml) | 22.0 ± 8.3 | 16.7 ± 7.0 | <0.001** |
| HOMA-IR | 4.8 ± 1.9 | 3.1 ± 1.4 | <0.001** |
| Adropin (ng/ml) | 2.64 ± 0.93 | 3.57 ± 0.95 | <0.001** |
| RHI | 1.70 ± 0.37 | 1.84 ± 0.29 | <0.001** |
| VO2peak,ml/kg/min | 26.7 ± 1.8 | 29.8 ± 1.7 | <0.01** |

Note: AST: aspartate aminotransferase; ALT: alanine aminotransferase; BMI: body mass index; BUN: blood urea nitrogen; DBP: diastolic blood pressure; Glu: fasting glucose; HDL-C: high density lipoprotein; HOMA-IR: insulin resistance index; LDL-C: low density lipoprotein; RHI: reactive hyperemia index; SBP: systolic blood pressure; TC: total cholesterol; TG: triglyceride; WHR**:** waist-to-hip ratio. Data are presented as mean ± standard deviation, * p<0.05, * * p<0.01

**Table S2 Comparison of parameters between before and after exercise intervention in the weight gain group**

|  | Before intervention (n = 8) | After intervention  (n = 8) | P value |
| --- | --- | --- | --- |
| Male (n, %) | 4(50%) | 4(50%) | - |
| Height (cm) | 165.8 ± 6.8 | 166.5 ± 7.2 | 0.10 |
| Weight (kg) | 88.5± 6.1 | 90.0 ± 7.0 | 0.037 |
| BMI (kg/m^2^) | 32.3 ± 3.0 | 32.6 ± 3.3 | 0.240 |
| SBP (mmHg) | 127.1 ± 10.6 | 121.6 ±9.0 | 0.118 |
| DBP (mmHg) | 70.3 ± 8.0 | 72.1 ± 7.2 | 0.704 |
| Fat mass (kg) | 32.6 ± 8.7 | 30.1 ± 11.3 | 0.052 |
| Total bilirubin (μmol/l) | 10.6 ± 3.6 | 9.7 ± 4.7 | 0.226 |
| AST (μmol/l /l) | 25.5 ± 13.7 | 19.9 ± 10.9 | 0.042* |
| ALT (μmol/l /l) | 21.1 ±6.5 | 18.9 ± 4.7 | 0.102 |
| BUN (mmol/l) | 4.3 ± 0.9 | 4.1 ± 1.0 | 0.224 |
| Creatinine (μmol/l) | 70.5 ± 10.8 | 69.5 ± 14.7 | 0.675 |
| Uric acid (μmol/l /l) | 425.2 ± 68.5 | 430.8 ± 91.9 | 0.805 |
| TC (mmol/l) | 3.69 ± 0.47 | 3.67 ± 0.56 | 0.711 |
| TG (mmol/l) | 1.42 ± 0.86 | 1.58 ± 1.22 | 0.427 |
| HDL-C (mmol/l) | 1.17 ± 0.20 | 1.46 ± 0.24 | <0.001** |
| LDL-C (mmol/l) | 1.93 ± 0.25 | 1.75 ± 0.24 | <0.001** |
| Glu (mmol/l) | 4.90 ± 0.42 | 4.15 ± 0.38 | <0.001** |
| Fasting insulin (μUI/ml) | 23.6 ± 9.2 | 20.0 ± 5.9 | 0.262 |
| HOMA-IR | 5.2± 2.1 | 3.7 ± 1.1 | 0.052 |
| Adropin (ng/ml) | 2.71 ±1.24 | 4.10 ± 1.78 | 0.004** |
| RHI | 1.64 ± 0.22 | 1.77 ± 0.22 | 0.020* |
| Peak VO2, ml/kg/min | 27.3 ± 1.9 | 30.3 ± 2.0 | <0.05* |

Note: AST: aspartate aminotransferase; ALT: alanine aminotransferase; BUN: blood urea nitrogen; DBP: diastolic blood pressure; Glu: fasting glucose; HDL-C: high density lipoprotein; HOMA-IR: insulin resistance index; LDL-C: low density lipoprotein; RHI: reactive hyperemia index; SBP: systolic blood pressure; TC: total cholesterol; TG: triglyceride. Data are presented as mean ± standard deviation, * p<0.05, * * p<0.01

| **Table S3. The Pearson correlation analysis of serum adropin level and the clinical data** | | |
| --- | --- | --- |
|
|  | Pearson correlation | P value |
| Age (Year) | 0.083 | 0.51 |
| SBP (mmHg) | -0.118 | 0.35 |
| DBP (mmHg) | -0.272 | 0.029* |
| Weight (kg) | -0.35 | 0.004** |
| BMI (kg/m^2^) | -0.248 | 0.046* |
| WHR | -0.335 | 0.008** |
| Fat mass (kg) | -0.235 | 0.064 |
| Total bilirubin (μmol/l) | 0.014 | 0.91 |
| AST (μmol/l /l) | -0.313 | 0.011* |
| ALT (μmol/l /l) | -0.174 | 0.166 |
| BUN (mmol/l) | -0.058 | 0.647 |
| Creatinine (μmol/l ) | -0.165 | 0.189 |
| Uric acid (μmol/l /l) | -0.424 | <0.001** |
| TC (mmol/l) | 0.031 | 0.804 |
| TG (mmol/l) | -0.316 | 0.01* |
| HDL-C (mmol/l) | 0.389 | 0.001** |
| LDL-C (mmol/l) | -0.078 | 0.536 |
| Glu (μUI/ml) | -0.266 | 0.032* |
| Fasting insulin (μUI/ml) | -0.41 | 0.001** |
| HOMA-IR | -0.44 | <0.001** |
| RHI | 0.32 | 0.009** |

Note: AST: aspartate aminotransferase; ALT: alanine aminotransferase; BMI: body mass index; BUN: blood urea nitrogen; DBP: diastolic blood pressure; Glu: fasting glucose; HDL-C: high density lipoprotein; HOMA-IR: insulin resistance index; LDL-C: low density lipoprotein; RHI: reactive hyperemia index; SBP: systolic blood pressure; TC: total cholesterol; TG: triglyceride; WHR**:** waist-to-hip ratio. Data are presented as mean ± standard deviation, * p<0.05, * * p<0.01

**Table S4.** Results of multiple linear regression analysis of serum adropin

| Model | Non standardized coefficient | | Standard coefficient | T | P |
| --- | --- | --- | --- | --- | --- |
|  | B | standard error |  |  |  |
| (constant) | 2.299 | 0.590 | / | 13.470 | <0.01** |
| HOMA-IR | -0.170 | 0.052 | -0.376 | -3.301 | <0.01** |
| HDL | 1.099 | 0.420 | 0.298 | 2.620 | 0.011* |

Note: HDL-C: high density lipoprotein; HOMA-IR: insulin resistance index. * p<0.05, * * p<0.01

| **Table S5 Pearson related analysis results of ΔRHI and clinical data in obese adolescents before and after exercise intervention** | | |
| --- | --- | --- |
|
|  | r | p |
| ΔSBP (mmHg) | 0.317 | 0.034* |
| ΔDBP (mmHg) | -0.137 | 0.370 |
| ΔWeight (kg) | 0.042 | 0.786 |
| ΔBMI (kg/m^2^) | 0.047 | 0.759 |
| ΔFat mass (kg) | -0.089 | 0.561 |
| ΔTotal bilirubin (μmol/l) | 0.05 | 0.745 |
| ΔAST (μmol/l /l) | 0.005 | 0.971 |
| ΔALT (μmol/l /l) | 0.025 | 0.872 |
| ΔBUN (mmol/l) | -0.043 | 0.781 |
| ΔCreatinine (μmol/l ) | 0.322 | 0.031* |
| ΔUric acid (μmol/l /l) | 0.195 | 0.20 |
| ΔTC (mmol/l) | 0.235 | 0.121 |
| ΔTG (mmol/l) | 0.088 | 0.564 |
| ΔHDL-C (mmol/l) | 0.083 | 0.589 |
| ΔLDL-C (mmol/l) | 0.09 | 0.559 |
| ΔGlu (mmol/l) | -0.093 | 0.542 |
| ΔFasting insulin (μUI/ml) | -0.082 | 0.593 |
| ΔHOMA-IR | -0.154 | 0.314 |
| ΔAdropin（ng/ml） | 0.445 | 0.002** |

Note: AST: aspartate aminotransferase; ALT: alanine aminotransferase; BMI: body mass index; BUN: blood urea nitrogen; DBP: diastolic blood pressure; Glu: fasting glucose; HDL-C: high density lipoprotein; HOMA-IR: insulin resistance index; LDL-C: low density lipoprotein; SBP: systolic blood pressure; TC: total cholesterol; TG: triglyceride; * p<0.05, * * p<0.01

**Figure**


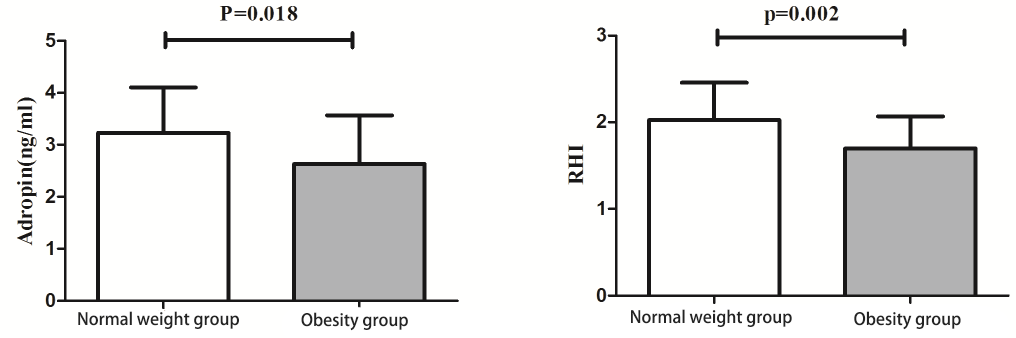
**Figure S1 Comparison of serum adropin concentration and vascular reactive congestion index (RHI) in normal weight group and obesity group**


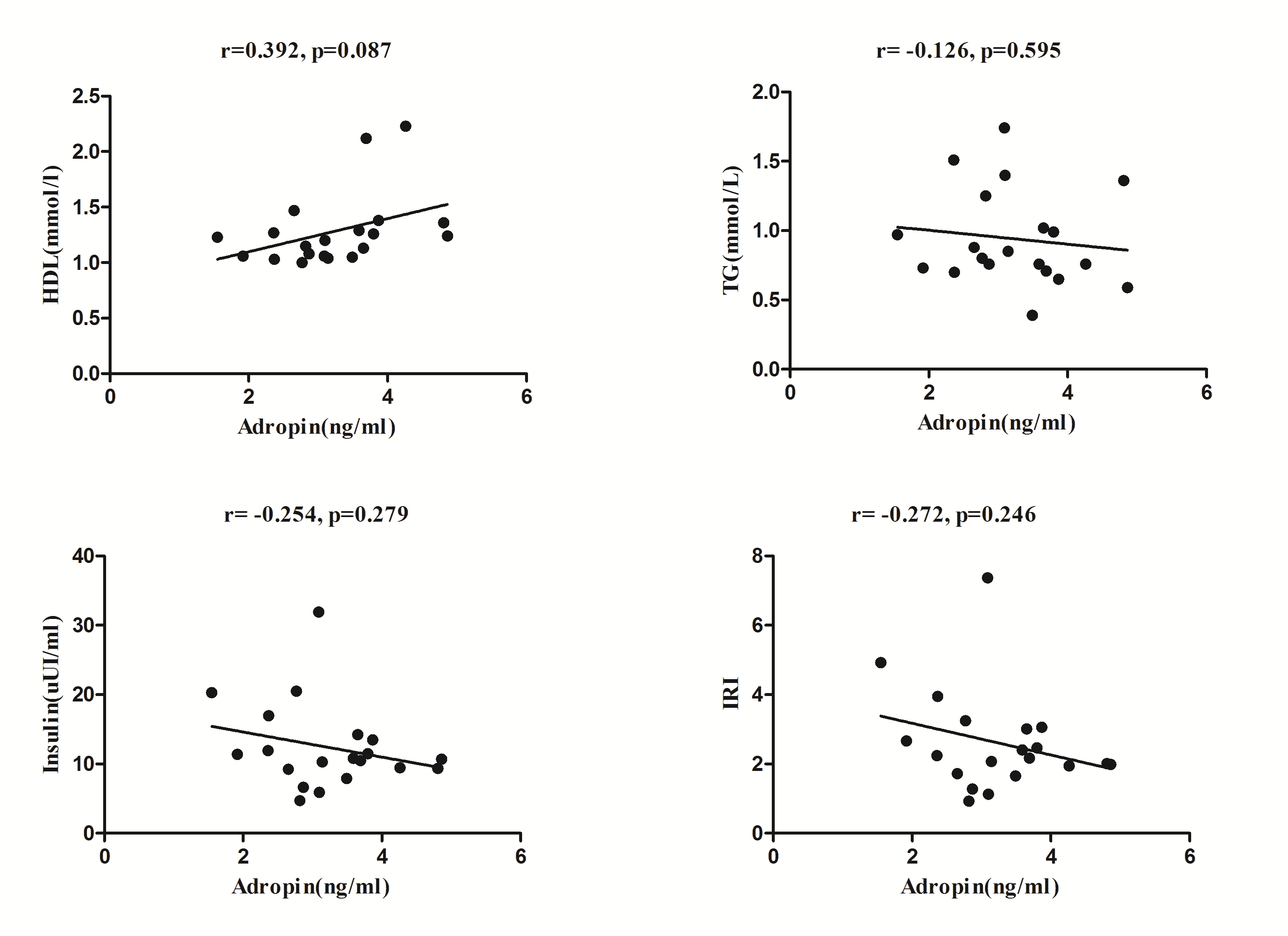


**Figure S2 The pearson correlation analysis of serum adropin level and TG, HDL-C, insulin, and RHI in control group**


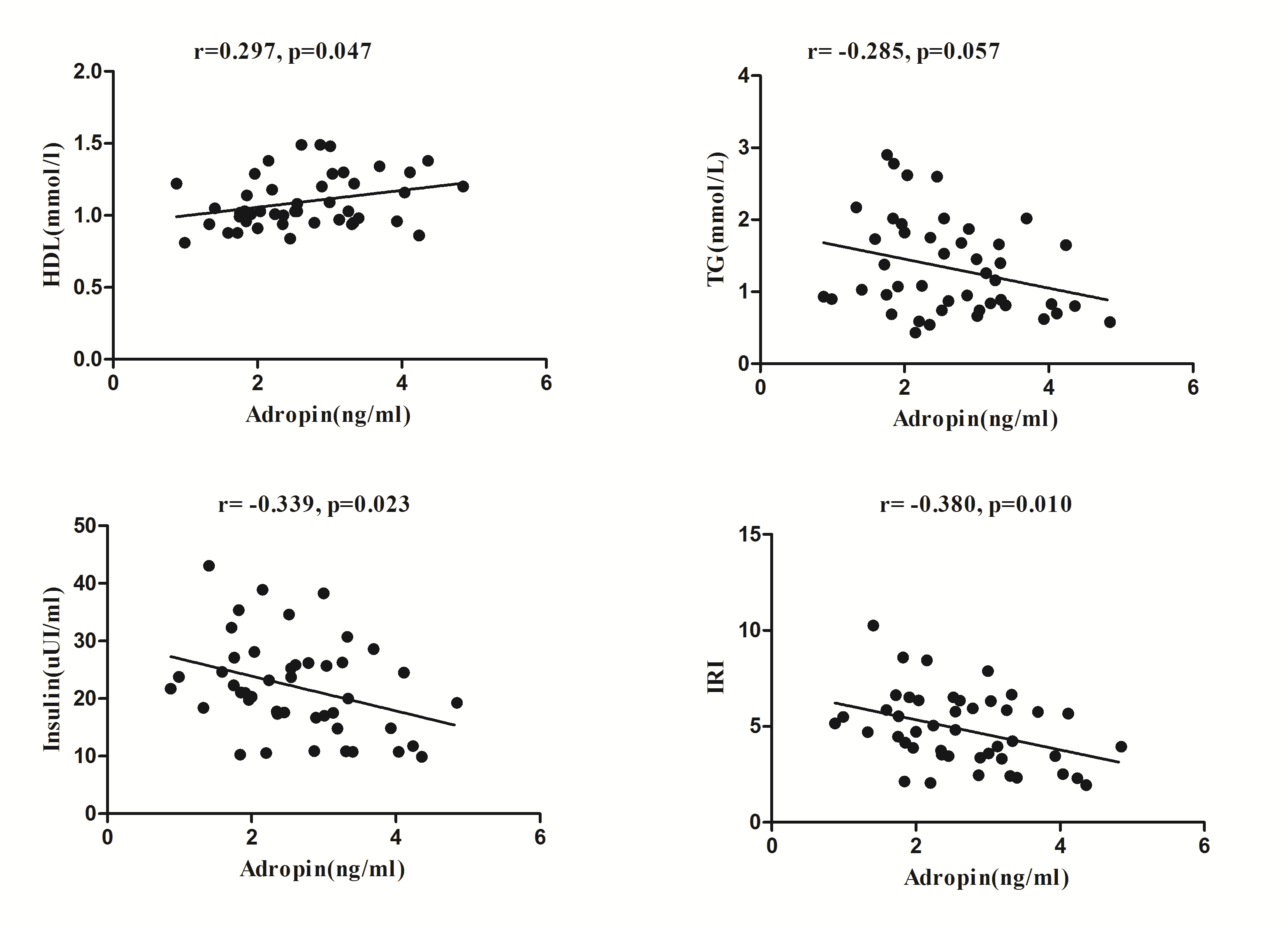


**Figure S3 The pearson correlation analysis of serum adropin level and TG, HDL-C, insulin, and RHI in obesity group**
